# Enhancing sparse representation of color images by cross channel transformation

**DOI:** 10.1371/journal.pone.0279917

**Published:** 2023-01-26

**Authors:** Laura Rebollo-Neira, Aurelien Inacio

**Affiliations:** 1 Mathematics Department, Aston University B4 7ET, Birmingham, United Kingdom; 2 Aurelien Inacio, ENSIIE, Paris, France; Dartmouth College Geisel School of Medicine, UNITED STATES

## Abstract

Transformations for enhancing sparsity in the approximation of color images by 2D atomic decomposition are discussed. The sparsity is firstly considered with respect to the most significant coefficients in the wavelet decomposition of the color image. The discrete cosine transform is singled out as an effective 3 point transformation for this purpose. The enhanced feature is further exploited by approximating the transformed arrays using an effective greedy strategy with a separable highly redundant dictionary. The relevance of the achieved sparsity is illustrated by a simple encoding procedure. On typical test images the compression at high quality recovery is shown to significantly improve upon JPEG and WebP formats.

## 1 Introduction

In the signal processing field sparse representation usually refers to the approximation of a signal as superposition of elementary components, called atoms, which are members of a large redundant set, called a dictionary [1]. The superposition, termed atomic decomposition, aims at approximating the signal involving as few atoms as possible [1–4]. Sparsity is also relevant to data collection. Within the emerging theory of sampling known as *compressive sensing* (CS) this property is key for reconstructing signals from a reduced number of measures [5–7]. In particular, distributed compressive sensing (DCS) algorithms exploit inter signal correlation structure for multiple signal recovery [8].

Sparse image representation using redundant dictionaries has been considered in numerous works e.g. [9–11] and in the context of applications such as image restoration [12–14], feature extraction [15–18] and super resolution [19–22].

The sparsity property of some types of 3D images benefits from 3D processing [23–26]. In particular most RGB (Red-Green-Blue) images admit a sparser atomic decomposition if approximated by 3D atoms [27]. Within the 3D framework the gain in sparsity comes at expenses of computational cost though.

The purpose of this work is to show that the application of a transformation across the direction of the colors improves sparsity in the wavelet domain representation of the 2D color channels. The relevance of this feature is demonstrated by a simple encoding procedure rendering good compression results in comparison to commonly used image compression standards.

The work is organized as follows: Sec. 2 introduces the mathematical notation. Sec. 3 compares several cross color transformations enhancing sparsity. A numerical example on a large data set is used to illustrate the suitability of the dct for that purpose. Sec. 4 demonstrates the gain in sparsity obtained by the atomic decomposition of color images when the dct is applied across the RGB channels. Sec. 5 illustrates the relevance of the approach to image compression with high quality recovery. The conclusions are summarized in Sec. 6.

## 2 Mathematical notation

Throughout the paper we use the following notational convention. R represents the set of real numbers. Boldface letters indicate Euclidean vectors, 2D and 3D arrays. Standard mathematical fonts indicate components, e.g., $d\in R^{N}$ is a vector of components d(i)∈R,i=1,…,N. The elements of a 2D array $I\in R^{L_{x}\times L_{y}}$ are indicated as *I*(*i*, *j*), *i* = 1, …, *L*_*x*_, *j* = 1, …, *L*_*y*_ and the color channels of $I\in R^{L_{x}\times L_{y}\times 3}$ as *I*(:, :, *z*), *z* = 1, 2, 3. The transpose of a matrix, **G** say, is indicated as **G**^⊤^. A set of, say *M*, color images is represented by the arrays $I\left\{m\right\}\in R^{L_{x}\times L_{y}\times 3},m=1,\ldots ,M.$

The inner product between arrays in $R^{L_{x}\times L_{y}}$ is given by the Frobenius inner product 〈⋅, ⋅〉_F_ as

$${\left\langle G,I\right\rangle }_{F}=\sum_{i=1}^{L_{x}}\sum_{j=1}^{L_{y}}G\left(i,j\right)I\left(i,j\right).$$

Consequently, the Frobenius norm ‖⋅‖_F_ is calculated as

$${∥G∥}_{F}=\sqrt{\sum_{i=1}^{L_{x}}\sum_{j=1}^{L_{y}}G{\left(i,j\right)}^{2}}.$$

The inner produce between arrays in $R^{N}$ is given by the Euclidean inner product 〈⋅, ⋅〉 as $\langle d,g\rangle =\sum_{i=1}^{N}d\left(i\right)g\left(i\right).$

## 3 Cross color transformations

Given a color image *I*(*i*, *j*, *z*), *i* = 1, …, *L*_*x*_, *j* = 1, …, *L*_*y*_, *z* = 1, 2, 3 the processing of the 3 channels can be realized either in the pixel/intensity or in the wavelet domain. Since the representation of most images is sparser in the wavelet domain [27–30] we approximate in that domain and reconstruct the approximated image by the inverse wavelet transform. Thus, using a 3 × 3 matrix **T**, we construct the transformed arrays $U\in R^{L_{x}\times L_{y}\times 3}$ and $W\in R^{L_{x}\times L_{y}\times 3}$ as follows

$$\begin{array}{ccc} U(:,:,z) & \;\;= & \;\;\sum_{l=1}^{3}I\left(:,:,l\right)T\left(l,z\right),\;z=1,2,3. \end{array}$$ (1)

$$\begin{array}{ccc} W(:,:,z) & \;\;=\;\; & \text{dwt}(U(:,:,z)),\;\;\;\;\;\;\;\;z=1,2,3, \end{array}$$ (2)

where **dwt** indicates the 2D wavelet transform. For the transformation **T** we consider the following cases

(i) The dct.
(ii) The reversible YCbCr color space transform.
(iii) The principal components (PC) transform.
(iv) A transformation learned from an independent set of images.

The dct is given by the matrix

$$(\begin{array}{ccc} \frac{1}{\sqrt{3}} & \frac{\sqrt{2}}{\sqrt{3}}\text{cos}\left(\frac{\pi }{6}\right) & \frac{\sqrt{2}}{\sqrt{3}}\text{cos}\left(\frac{\pi }{3}\right)\\ \frac{1}{\sqrt{3}} & 0 & \frac{\sqrt{2}}{\sqrt{3}}\text{cos}\left(\pi \right)\\ \frac{1}{\sqrt{3}} & \frac{\sqrt{2}}{\sqrt{3}}\text{cos}\left(\frac{5\pi }{6}\right) & \frac{\sqrt{2}}{\sqrt{3}}\text{cos}\left(\frac{5\pi }{3}\right) \end{array}),$$

whereas the YCbCr color space transform is given by the matrix below [31]

$$(\begin{array}{ccc} 0.299 & -0.169 & 0.5\\ 0.587 & -0.331 & -0.419\\ 0.114 & 0.5 & -0.0813 \end{array}).$$

The columns of the principal components transform are the normalized eigenvectors of covariance matrix of the RGB pixels. Thus, the transformation is image dependent and optimal with respect to decorrelating the color channels.

As a first test, the approximation of the transformed channels is realized by keeping a fixed number of the largest absolute value entries, and setting the others equal to zero. In relation to this, for an image of size *L*_*x*_ × *L*_*y*_ × 3 we define the Sparsity Ratio (SR) as follows

$$\begin{array}{c} \text{SR}=\frac{L_{x}\cdot L_{y}\cdot 3}{\text{Number}\;\text{of}\;\text{nonzero}\;\text{entries}\;\text{in}\;\text{the}\;\text{three}\;\text{channels}}. \end{array}$$ (3)

The quality of a reconstructed image $\tilde{I}$, with respect to the original 8-bit image **I**, is compared using the Peak Signal-to-Noise Ratio (PSNR)

$$\text{PSNR}=10{\text{log}}_{10}(\frac{255^{2}}{\text{MSE}}),\;\text{with}\;\text{MSE}=\frac{1}{L_{x}\cdot L_{y}\cdot 3}\sum_{i,j,z=1}^{L_{x},L_{y},3}{\left(I\left(i,j,z\right)-\tilde{I}\left(i,j,z\right)\right)}^{2}.$$

For the numerical examples below the transformation corresponding to case (iv) is learned from a set of images **I**{*m*}, *m* = 1, …, *M* all of the same size. Starting from an invertible 3 × 3 matrix **T**^*k*^, with *k* = 1, the learning algorithm proceeds through the following instructions.

1. Use **T**^*k*^ to transform the arrays **I**{*m*}→**U**^*k*^{*m*}→**W**^*k*^{*m*} as in (1) and (2).
2. Approximate each transformed array **W**^*k*^{*m*} to obtain ${\tilde{W}}^{k}\left\{m\right\}$ by keeping the largest *K* absolute value entries.
3. Apply the inverse 2D wavelet transform **idwt** to reconstruct the approximated arrays ${\tilde{U}}^{k}\left\{m\right\},\;m=1,\ldots ,M$ as
  $$\begin{array}{ccc} {\tilde{U}}^{k}\left\{m\right\}\left(:,:,z\right) & \;\;\;=\;\;\; & \text{idwt}({\tilde{W}}^{k}\left\{m\right\}\left(:,:,z\right)),\;\;z=1,2,3. \end{array}$$
4. Use the original images **I**{*m*}, *m* = 1, …, *M* to find **G** = **T**^−1^ by least squares fitting, i.e
  $$G^{✱}=\underset{\begin{array}{c} G(z,l)\\ z,l=1,2,3 \end{array}}{\text{arg}\;\text{min}}E\left(G\right),$$
  where
  $$E\left(G\right)=\sum_{m=1}^{M}\sum_{i,j,l=1}^{L_{x},L_{y},3}{\left(I\left\{m\right\}\left(i,j,l\right)-\tilde{I}\left\{m\right\}\left(i,j,l\right)\right)}^{2}$$
  with
  $$\tilde{I}\left\{m\right\}\left(i,j,l\right))=\sum_{z=1}^{3}{\tilde{U}}^{k}\left\{m\right\}\left(i,j,z\right)G\left(z,l\right).$$
5. While E decreases, or the maximum number of allowed iterations has not been reached, set *k* → *k* + 1, **T**^*k*^ = (G*)^−1^ and repeat steps 1)–5).

Given the arrays ${\tilde{U}}^{k}\left\{m\right\},\;m=1,\ldots ,M$ the least squares problem for determining the transformation **T**^*k*^ has a unique solution. However, the joint optimization with respect to the arrays ${\tilde{U}}^{k}\left\{m\right\},m=1,\ldots ,M$ *and* the transformation **T**^*k*^ is not convex. Hence, the algorithm’s outcome depends on the initial value.

**Fig 1:**
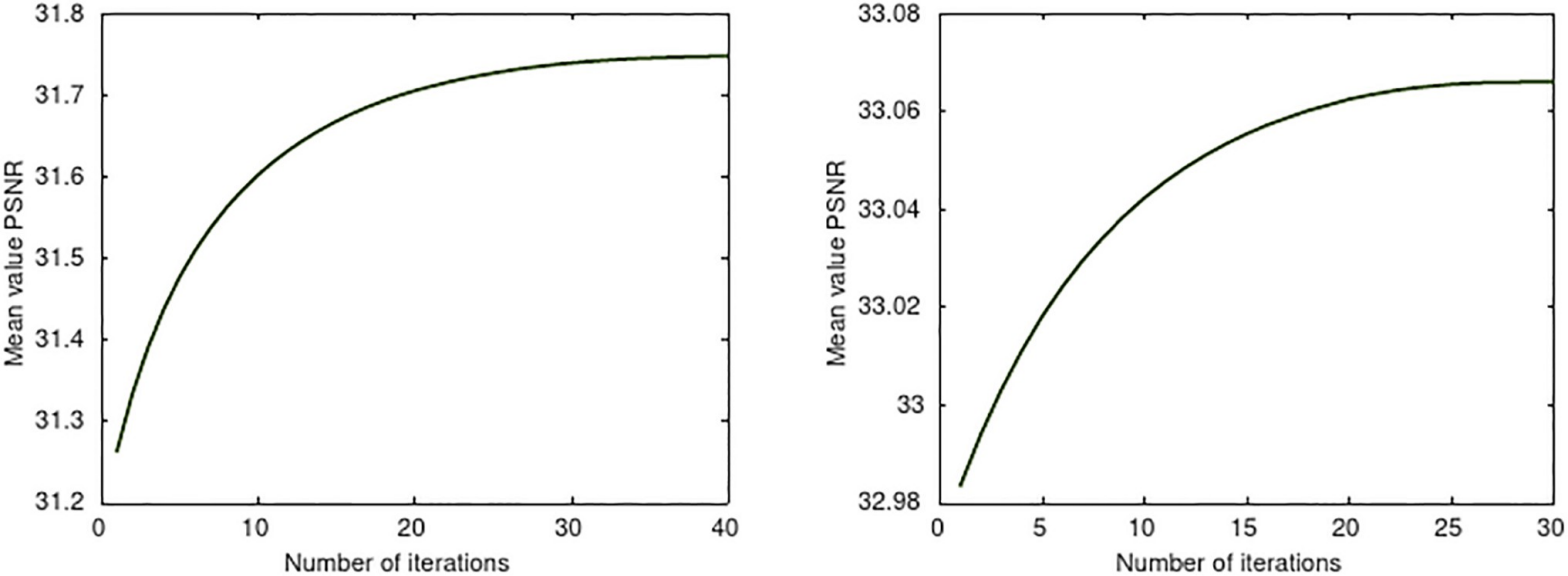
Learning curves for the transformation (iv) corresponding to two different random orthogonal transforms initializing the process. The mean value PSNR with respect to the 100 images in the training set corresponds to SR = 15 for all the images.

The transformation (iv) used in the numerical examples of Secs. 3.1 and 4.1 has been learned from *M* = 100 images, all of size 384 × 512 × 3, from the UCID data set [32], which contains images of buildings, places and cars. The learning curves for two random orthonormal initializations is shown in Fig 1.

It is worth mentioning that, as shown in Fig 1, learning is richer when starting comparatively distant from a local minimum (left graph in Fig 1). However, since the convergence is to a local minimum other random initializations, even if generating less learning, may produce better results (right graph in Fig 1).

The aim of the next numerical test is to demonstrate the effect on the SR (c.f. (3)) produced by the above (i)–(iv) transformations across the color channels.

### 3.1 Numerical test I

**Fig 2:**
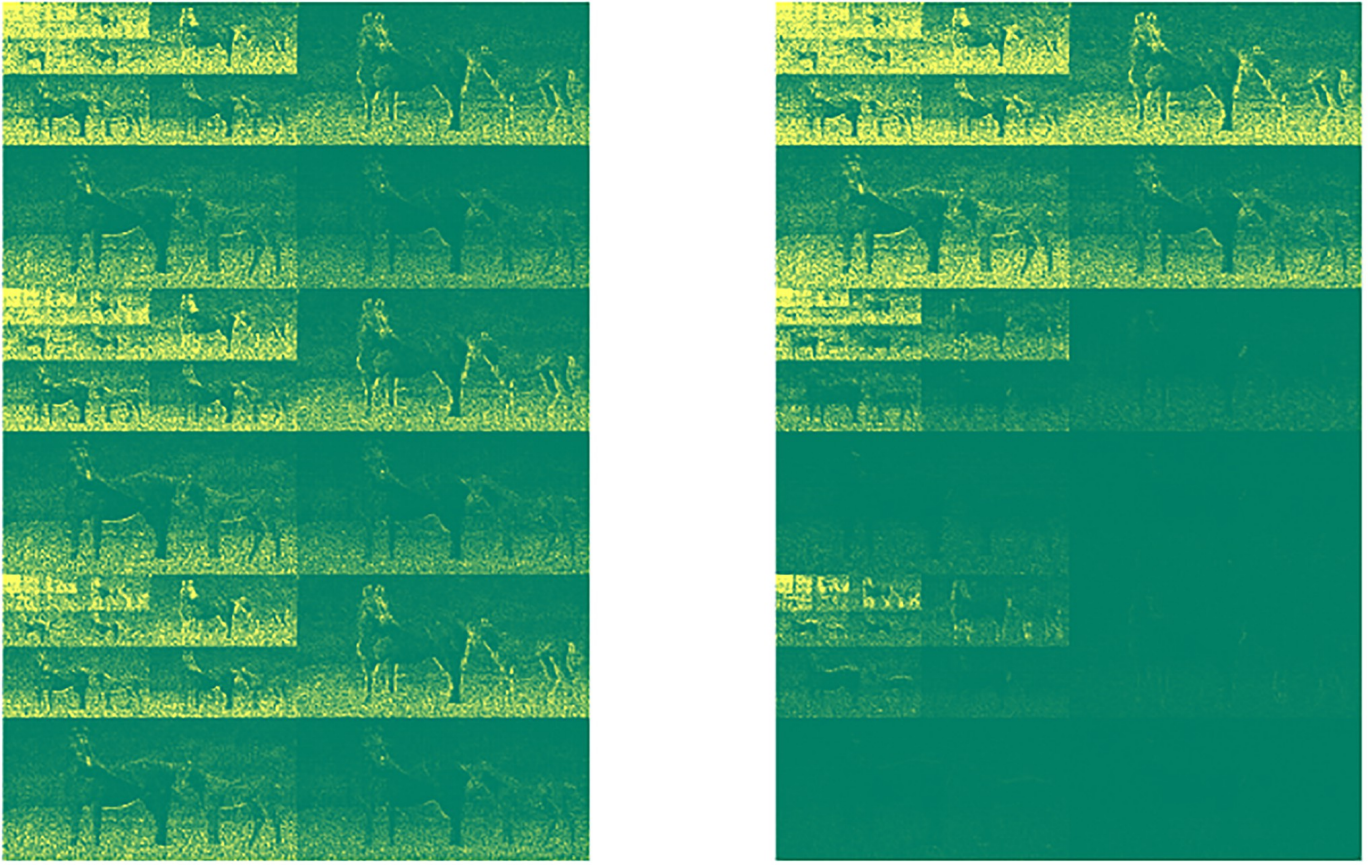
Magnitude of the entries in the array W constructed as in (2) from the image of Fig 3, with T the dct (right graph) and without T (left graph).

**Fig 3:**
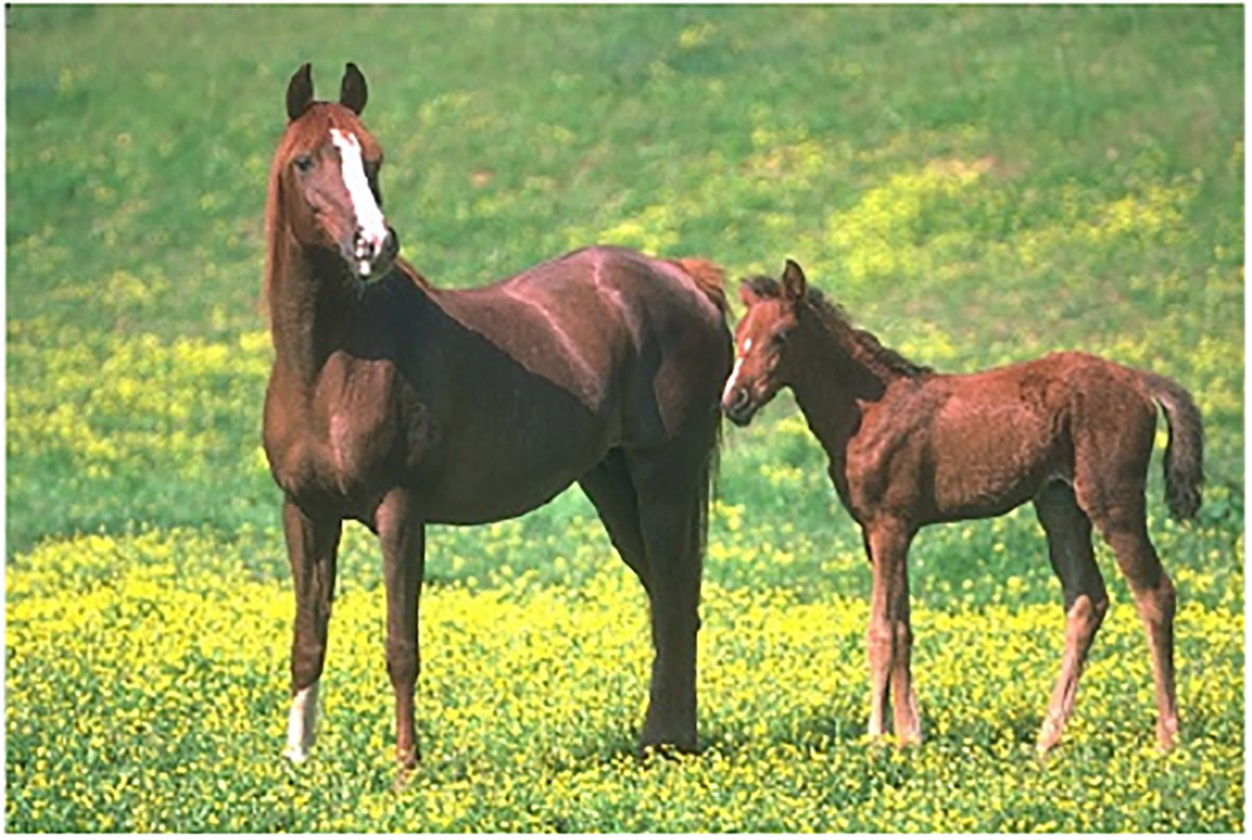
One of the RGB images in the Berkeley’s data set.

Using the whole Berkeley data set [33], consisting of 300 images all of size 321 × 481 × 3 we proceed with each image as in (1) and (2). The **dwt** corresponds to the Cohen-Daubechies-Feauveau 9/7 (CDF97) wavelet family. Fig 2 shows the transformed channels of the image in Fig 3, including the dct transformation across channels (right graph) and without **T** transformation (left graph). As noticeable in the figure, the effect of the dct is to re-distribute the intensity in the color channels by transferring values between channels.

For the numerical test the approximations are realized fixing SR = 20 and SR = 10. The reconstructed images are obtained for the approximated arrays $\tilde{W}$ as

$$\begin{array}{ccc} \tilde{U}\left(:,:,z\right) & = & \text{idwt}(\tilde{W}\left(:,:,z\right)),\;\;\;\;\;\;\;\;z=1,2,3 \end{array}$$ (4)

$$\begin{array}{ccc} \tilde{I}\left(:,:,z\right) & = & \;\;\;\sum_{l=1}^{3}\tilde{U}\left(:,:,l\right)\left(T^{-1}\left(z,l\right)\right),\;z=1,2,3 \end{array}$$ (5)

where **T**^−1^ is the inverse of **T**. When no transformation **T** is considered the image is reconstructed directly from (4).

The 2nd and 4th columns of Table 1 show the mean value PSNR ($\bar{\text{PSNR}}$), with respect to the whole data set, for SR = 20 and SR = 10 respectively, corresponding to the transformations **T** listed in the 1st column of the table. The 3rd and 5th columns give the standard deviations (std).

**Table 1:** Mean value PSNR, with respect to the 300 images in the Berkeley data set. The approximation of each image is realized by setting the least significant entries in the arrays **W**{*m*} = 1, …, 300 equal zero, in order to obtain SR = 20 for all the images (2nd column) and SR = 10 for all the images (4th column).

Transf.	$\bar{\text{PSNR}}$	std	$\bar{\text{PSNR}}$	std
(i) dct	33.9	5.0	38.9	5.1
(ii) YCbCr	33.7	5.0	38.7	5.1
(iii) PC	33.7	4.9	38.4	5.0
(iv) Learned	33.7	4.9	38.7	5.0
(v) No transf.	29.7	4.8	32.9	5.2

While Table 1 shows that all (i)–(iv) transformations render equivalent superior results, with respect to not applying a transformation (case (v)), the dct slightly exceeds the others. Case (iv) refers to the best result achieved when initializing the learning algorithm with 500 different random orthonormal transformations. When initializing the algorithm with transformations (i) and (ii) it appears that each of these transformations is close to a local minimizer of the method. This stems from the fact that such initializations do not generate significant learning.

The common feature of most of the 300 images in the data set used in this numerical example is the correlation property of the three color channels. This property is assessed by the correlation coefficients

$$\begin{array}{c} r\left(z\right)=\frac{\sum_{i=1}^{L_{x}}\sum_{j=1}^{L_{y}}\Gamma \left(i,j,z\right)\Gamma \left(i,j,z+1\right)}{\sigma (z)\sigma (z+1)},\;z=1,2,\;r\left(3\right)=\frac{\sum_{i=1}^{L_{x}}\sum_{j=1}^{L_{y}}\Gamma \left(i,j,1\right)\Gamma \left(i,j,3\right)}{\sigma (1)\sigma (3)}, \end{array}$$

where

$$\begin{array}{c} \Gamma \left(i,j,z\right)=\left(I\left(i,j,z\right)-\bar{I(:,:,z)}\right),\;\;\sigma \left(z\right)=\sqrt{\sum_{i=1}^{L_{x}}\sum_{j=1}^{L_{y}}{\left(I\left(i,j,z\right)-\bar{I(:,:,z)}\right)}^{2}}, \end{array}$$ (8)

and $\bar{I(:,:,z)}$ indicates the mean value of channel *z*.

The range of the correlation coefficient *r*(*z*), *z* = 1, 2, 3 for the images in the data set can be better estimated using the Fisher transform [34, 35] which is defined as follows

$$\zeta \left(z\right)=\frac{1}{2}\text{ln}(\frac{r(z)+1}{r(z)-1}),\;z=1,2,3.$$

**Fig 4:**
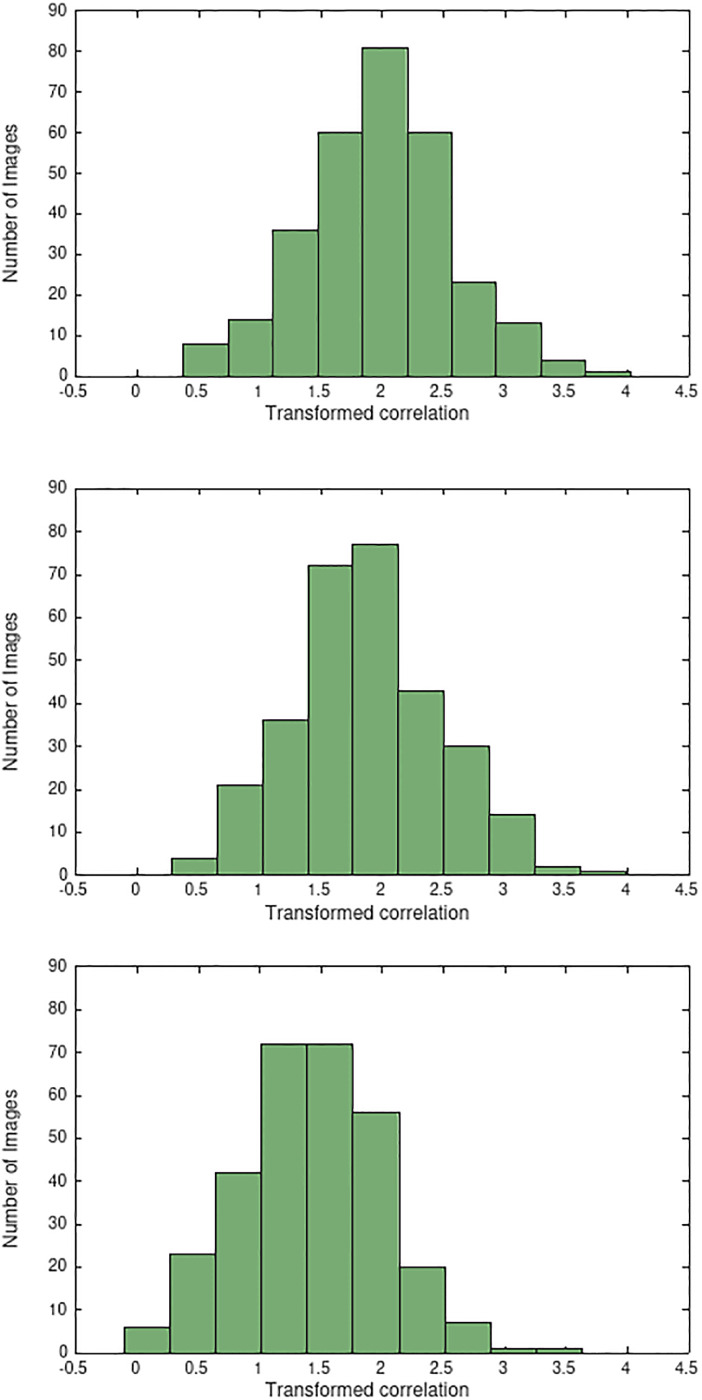
Histograms of the transformed correlation coefficients between the RGB channels: *ζ*(1) (top graph), *ζ*(2) (middle graph), and *ζ*(3) (bottom graph).

As seen in the graphs of Fig 4, the histograms of the transformed coefficients *ζ*(*z*), *z* = 1, 2, 3 resemble normal distributions. Hence, the confidence intervals can be well estimated in this domain. Once that is done, the intervals for the correlation coefficients are retrieved through the inverse transformation

$$r\left(z\right)=\frac{e^{2\zeta (z)}-1}{e^{2\zeta (z)}+1},\;z=1,2,3.$$

Table 2 gives the range of the correlation coefficients concerning approximately 68% and 95% of the images in the data set.

**Table 2:** Intervals for the correlation coefficients between R and G channels, *r*(1), G and B channels, *r*(2), and R and B channels, *r*(3), involving approximately 68% and 95% of the images in the data set.

Percentage	*r*(1)-interval	*r*(2)-interval	*r*(3)-interval
68%	(0.8821, 0.9882)	(0.8559, 0.9854)	(0.6881, 0.9854)
95%	(0.6612, 0.9964)	(0.5968, 0.9955)	(0.2617, 0.9884)

In view of the high correlation between the color channels for most images in the data set it is surprising than the PC transformation (iii), which completely decorrelates the channels, does not overperform the other transformations, on the contrary. This feature has also been noticed in the context of bit allocation for subband color image compression [36].

## 4 Approximations by atomic decomposition

We have seen (c.f. Table 1) that by transformation of channels it is possible to gain quality when reducing nonzero entries in the channels. Now we discuss how to improve quality further by approximating the 2D arrays (2) by an atomic decomposition, other than just by neglecting their less significant entries. For the approximation to be successful it is important to use an appropriate dictionary. To this end, one possibility could be to learn the dictionary from training data [37–40]. However as demonstrated in previous works [27, 29, 30] a separable dictionary, which is easy to construct, is well suited for the purposes of achieving sparsity and delivers a fast implementation of the approach. Since we use that dictionary in the numerical examples, below we describe the method for constructing the atomic decomposition of the array **W** considering specifically a separable dictionary.

Firstly we concatenate the 3 planes *W*(:, :, *z*), *z* = 1, 2, 3 into an extended 2D array $W^{\prime }\in R^{3L_{x}\times L_{y}}$ and divide this array in small blocks $W_{q}^{\prime },\;q=1,\ldots ,Q$. Without loss of generality the blocks are assumed to be square of size *N*_*b*_ × *N*_*b*_ say, and are approximated using separable dictionaries $D^{x}=\{d_{n}^{x}\in R^{N_{b}},\;∥d_{n}^{x}{{∥}_{2}=1\}}_{n=1}^{M_{b}}$ and $D^{y}=\{d_{m}^{y}\in R^{N_{b}},\;∥d_{n}^{y}{{∥}_{2}=1\}}_{m=1}^{M_{b}}$.

For *q* = 1, …, *Q* every element ${W^{\prime }}_{q}\in R^{N_{b}\times N_{b}}$ is approximated by an *atomic decomposition* as below:

$$\begin{array}{c} {W^{\prime }}_{q}^{k_{q}}=\sum_{n=1}^{k_{q}}c^{k_{q},q}\left(n\right)d_{\ell_{n}^{x,q}}^{x}{\left(d_{\ell_{n}^{y_{,}q}}^{y}\right)}^{T}, \end{array}$$ (6)

where $\ell_{n}^{y_{,}q}$ is the index in the set {1, 2, …, *M*_*b*_} corresponding to the label of the atom in the dictionary $D^{y}$ contributing to the *n*-th term in the approximation of the *q*-th block. The index $\ell_{n}^{x_{,}q}$ has the equivalent description. The assembling of the approximated blocks gives rise to the approximated array ${W^{\prime }}^{a}={\hat{\text{J}}}_{q=1}^{Q}{W^{\prime }}_{q}^{k_{q}}$, where $\hat{\text{J}}$ represents the assembling operation, i.e. the operation that retrieves the approximation ${W^{\prime }}^{a}\in R^{3L_{x}\times L_{y}}$ of the array $W^{\prime }\in R^{3L_{x}\times L_{y}}$ from the approximation of the blocks in the partition. The approximated array ${W^{\prime }}^{a}\in R^{3L_{x}\times L_{y}}$ is reshaped back into 3 planes, $W^{a}\left(:,:,z\right)\in R^{L_{x}\times L_{y}},\;z=1,2,3$, to be converted back to the approximated RGB intensity image as in (4) and (5).

The approximation of the partition ${W^{\prime }}_{q}\in R^{N_{b}\times N_{b}},\;q=1,\ldots ,Q$ is carried out iteratively as a two step process which selects i) the atoms in the atomic decomposition (6) and ii) the sequence in which the blocks in the partition are approximated. The procedure is called Hierarchized Block Wise (HBW) implementation of greedy pursuit strategies [28, 41]. For the selection of the atoms we apply the Orthogonal Matching Pursuit (OMP) approach [42] dedicated to 2D with separable dictionaries (OMP2D) [43]. Thus, the whole algorithm is termed HBW-OMP2D [28]. The method iterates as described below.

On setting *k*_*q*_ = 0 and $R_{q}^{0}={W^{\prime }}_{q}\in R^{N_{b}\times N_{b}}$ at iteration *k*_*q*_ + 1 the algorithm selects the indices $\ell_{k_{q}+1}^{x,q}$ and $\ell_{k_{q}+1}^{y,q}$, as follows:

$$\begin{array}{c} \ell_{k_{q}+1}^{x,q},\ell_{k_{q}+1}^{y,q}=\underset{\begin{array}{c} n=1,\ldots ,M_{b}\\ m=1,\ldots ,M_{b} \end{array}}{\text{arg}\;\text{max}}\left|{\left\langle d_{n}^{x},R_{q}^{k_{q}}d_{m}^{y}\right\rangle }_{F}\right|,\;\;\;q=1,\ldots ,Q, \end{array}$$ (7)

where $R_{q}^{k_{q}}={W^{\prime }}_{q}-{W^{\prime }}_{q}^{k_{q}}$. The calculation of ${W^{\prime }}_{q}^{k_{q}}$ is realized in order to minimize $∥R_{q}^{k_{q}}{∥}_{F}$, which is equivalent to finding the orthogonal projection onto the subspace spanned by the selected atoms ${\left\{A_{n}=d_{\ell_{n}^{x,q}}^{x}{\left(d_{\ell_{n}^{y,q}}^{y}\right)}^{T}\in R^{N_{b}\times N_{b}}\right\}}_{n=1}^{k_{q}}$. In our implementation, the calculation of the coefficients *c*^*q*^(*n*), *n* = 1, …, *k*_*q*_ in (6) is realized as

$$\begin{array}{c} c^{k_{q},q}\left(n\right)={\left\langle B_{n}^{k},{W^{\prime }}_{q}^{k_{q}}\right\rangle }_{F},\;n=1,\ldots ,k_{q}, \end{array}$$ (8)

where the set ${\left\{B_{n}^{k_{q}}\in R^{N_{b}\times N_{b}}\right\}}_{n=1}^{k_{q}}$ is biorthogonal to the set ${\left\{A_{n}\in R^{N_{b}\times N_{b}}\right\}}_{n=1}^{k_{q}}$ and needs to be upgraded and updated to account for each newly selected atom. Starting from $B_{1}^{1}=Q_{1}=A_{1}=d_{\ell_{1}^{x}}^{x}{\left(d_{\ell_{1}^{y}}^{y}\right)}^{⊤}$ the updating and upgrading is realized through the recursive equations [43, 44]:

$$\begin{array}{c} \begin{array}{cc} B_{n}^{k_{q}+1} & =B_{n}^{k_{q}}-B_{k_{q}+1}^{k_{q}+1}{\left\langle A_{k_{q}+1},B_{n}^{k}\right\rangle }_{F},\;n=1,\ldots ,k_{q}\\ B_{k_{q}+1}^{k_{q}+1} & =Q_{k_{q}+1}/{∥Q_{k_{q}+1}∥}_{F}^{2},\;\;\text{where}\\ Q_{k_{q}+1} & =A_{k_{q}+1}-\sum_{n=1}^{k_{q}}\frac{Q_{n}}{∥Q_{n}{∥}_{F}^{2}}{\left\langle Q_{n},A_{k_{q}+1}\right\rangle }_{F}, \end{array} \end{array}$$ (9)

with the additional re-orthogonalization step

$$\begin{array}{c} Q_{k_{q}+1}\leftarrow Q_{k_{q}+1}-\sum_{n=1}^{k_{q}}\frac{Q_{n}}{∥Q_{n}{∥}_{F}^{2}}.{\left\langle Q_{n},Q_{k_{q}+1}\right\rangle }_{F}. \end{array}$$ (10)

As discussed in [28, 41], for $\ell_{k_{q}+1}^{x,q}$ and $\ell_{k_{q}+1}^{y,q},\;q=1,\ldots ,Q$ the indices resulting from (7), the block to be approximated in the next iteration corresponds to the value *q*^⋆^ such that

$$q^{⋆}=\underset{q=1,\ldots ,Q}{\text{arg}\;\text{max}}|\langle d_{\ell_{k_{q}+1}^{x,q}}^{x},R_{q}^{k_{q}}\;d_{\ell_{k_{q}+1}^{y,q}}^{y}\rangle |.$$

The algorithm stops when the required total number of $K=\sum_{q=1}^{Q}k_{q}$ atoms has been selected. This number can be fixed using the SR, which is now calculated as

$$\begin{array}{c} \text{SR}=\frac{L_{x}\cdot L_{y}\cdot 3}{K}. \end{array}$$ (11)

**Remark 1**. *The above described implementation of HBW-OMP2D is very effective in terms of speed, but demanding in terms of memory (the partial outputs corresponding to all the blocks in the partition need to be stored at every iteration). An alternative implementation, termed HBW Self Projected Matching Pursuit (HBW-SPMP)* [29, 45], *would enable the application of the identical strategy to much larger images than the ones considered in this work*.

### 4.1 Numerical example II

**Fig 5:**
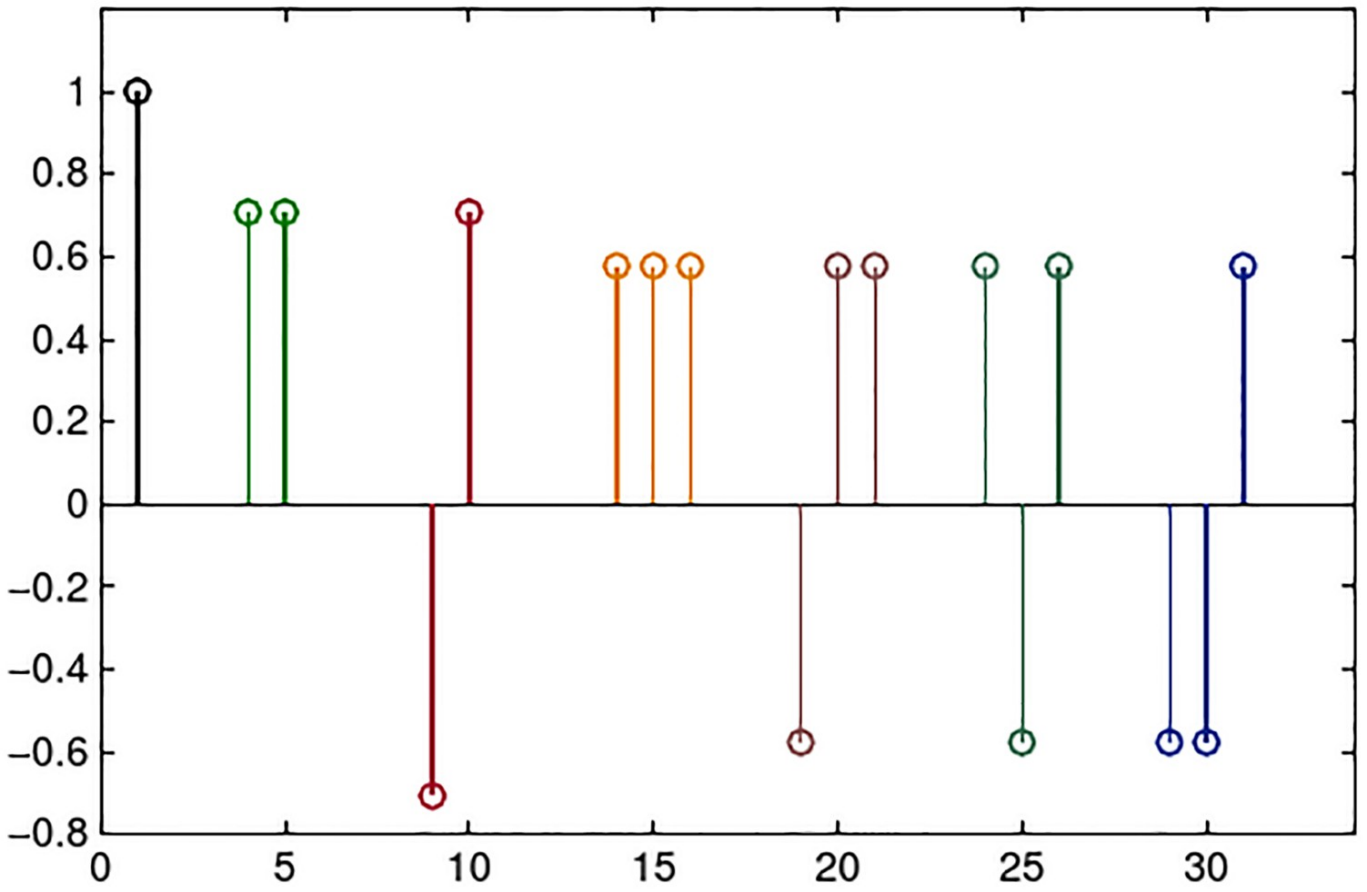
Prototypes (each in different color) that generate the dictionaries $D_{L}^{x}$ by sequential translations of one point.

For this and the next numerical example, we use a mixed dictionary consisting of two classes of sub-dictionaries of different nature:

I) The trigonometric dictionaries $D_{C}^{x}$ and $D_{S}^{x}$, defined below, for *i* = 1…, *N*_*b*_
  $$D_{C}^{x}={\left\{w_{c}\left(n\right)\text{cos}\frac{\pi (2i-1)(n-1)}{2M}\right\}}_{n=1}^{M_{x}},\;D_{S}^{x}={\left\{w_{s}\left(n\right)\text{sin}\frac{\pi (2i-1)(n)}{2M}q\right\}}_{n=1}^{M_{x}}.$$
  *w*_*c*_(*n*) and *w*_*s*_(*n*), *n* = 1, …, *M*_*x*_ are normalization factors.
II) The dictionary $D_{L}^{x}$, which is constructed by translation of the prototype atoms in Fig 5.

The mixed dictionary $D^{x}$ is built as $D^{x}=D_{C}^{x}\cup D_{S}^{x}\cup D_{L}^{x}$ and $D^{y}=D^{x}$.

Table 3 shows the improvement in $\bar{\text{PSNR}}$ achieved by atomic decompositions using the mixed dictionary for SR = 20 and SR = 10.

**Table 3:** Mean value PSNR, with respect to 300 images in the Berkeley data set, produced by 2D atomic decomposition of the arrays W{*m*} = 1, …, 300 in order to obtain SR = 20 (2nd and 6th column) and SR = 10 (4th and 8th column).

Block size	8 × 8				16 × 16			
Transf.	$\bar{\text{PSNR}}$	std	$\bar{\text{PSNR}}$	std	$\bar{\text{PSNR}}$	std	$\bar{\text{PSNR}}$	std
(i) dct	40.5	5.0	48.1	4.4	40.8	4.9	48.6	4.2
(ii) YCbCr	40.3	5.0	47.8	4.4	40.6	4.9	48.3	4.2
(iii) PC	39.6	4.8	46.3	4.5	39.9	4.8	46.7	4.5
(iv) Learned	40.3	4.9	47.4	4.3	40.5	4.9	47.8	4.2
(v) No transf.	34.0	5.2	39.1	5.6	34.1	5.2	39.3	5.5

Notice that while case (v), which does not include any **T** transformation, gives superior results than by disregarding entries (c.f. Table 1) when applying any of the transformations (i)–(iv) the results improve further. The PC transform, however, appears significantly less effective than the others. In addition to rendering the best results, the dct brings along the additional advantage of being orthonormal. Consequently, it does not magnify errors at the inversion step. Because of this, we single out the dct as the most convenient cross color transformation out of the four considered here.

## 5 Application to image compression

In order achieve compression by filing an atomic decomposition we need to address two issues. Namely, the quantization of the coefficients *c*_*q*_(*n*)*n* = 1, …, *k*_*q*_, *q* = 1, …, *Q* in (6) and the storage of the indices $\left(\ell_{n}^{x,q},\ell_{n}^{y,q}\right),n=1,\ldots ,k_{q},\;q=1,\ldots ,Q$. We tackle the matters by simple but effective procedures [30].

For *q* = 1, …, *Q* the absolute value coefficients |*c*_*q*_(*n*)|, *n* = 1, …, *k*_*q*_ are converted to integers through uniform quantization as follows

$$\begin{array}{c} c_{q}^{\Delta }\left(n\right)=\{\begin{array}{cc} \lceil \frac{|c_{q}\left(n\right)|-\theta }{\Delta }\rceil , & \text{if}\;|c_{q}\left(n\right)|\geq \theta \\ & 0\;\text{otherwise}. \end{array} \end{array}$$ (12)

The signs of the coefficient are encoded separately as a vector, **s**_*q*_, using a binary alphabet. Each pair of indices $\left(\ell_{n}^{x,q},\ell_{n}^{y,q}\right)$ corresponding to the atoms in the decompositions of the block $W_{q}^{\prime }$ is mapped into a single index *o*_*q*_(*n*). The set *o*_*q*_(1), …, *o*_*q*_(*k*_*q*_) is sorted in ascending order $o_{q}\left(n\right)\to {\tilde{o}}_{q}\left(n\right),\;n=1,\ldots ,k_{q}$ to take the differences $\delta_{q}\left(n\right)={\tilde{o}}_{q}\left(n\right)-{\tilde{o}}_{q}\left(n-1\right),\;n=2,\ldots ,k_{q}$ and construct the string of non-negative numbers ${\tilde{o}}_{q}\left(1\right),\delta_{q}\left(2\right),\ldots ,\delta_{q}\left(k_{q}\right)$. The order of the set ${\tilde{o}}_{q}\left(n\right),\;n=1,\ldots ,k_{q}$ induces order in the unsigned coefficients, $c_{q}^{\Delta }\left(n\right)\to {\tilde{c}}_{q}^{\Delta }\left(n\right)$, and in the corresponding signs $s_{q}\left(n\right)\to {\tilde{s}}_{q}\left(n\right)$.

For each *q* the number 0 is added at the end of the indices ${\tilde{o}}_{q}\left(n\right),\;n=1,\ldots ,k_{q}$ before concatenation, to be able to separate strings corresponding to different blocks. Each sequence of strings corresponding to *q* = 1, …, *Q* is concatenated and encoded using the off-the-shelf MATLAB function Huff06 [46], which implements Huffman coding.

The compression rate is given in bits-per-pixel (bpp) which is defined as

$$\text{bpp}=\frac{\text{Size}\;\text{of}\;\text{the}\;\text{file}\;\text{in}\;\text{bits}}{\text{Number}\;\text{of}\;\text{intensity}\;\text{pixels}\;\text{in}\;\text{a}\;\text{single}\;\text{channel}}.$$

At the reconstruction stage the indices $\left({\tilde{\ell }}_{n}^{x,q},{\tilde{\ell }}_{n}^{y,q}\right),\;n=1\;\ldots k_{q}$ are recovered from the string of differences *δ*_*q*_(*n*), *n* = 2, …, *k*_*q*_. The signs of the coefficients are read from the binary string. The quantized unsigned coefficients are read and transformed into real numbers as:

$$|{\tilde{c}}_{q}^{r}\left(n\right)|=\Delta \cdot {\tilde{c}}_{q}^{\Delta }\left(n\right)+\left(\theta -\Delta /2\right),\;n=1\;\ldots k_{q}.$$

The codec for reproducing the examples in the next sections has been made available on [47].

### 5.1 Numerical example III

**Fig 6:**
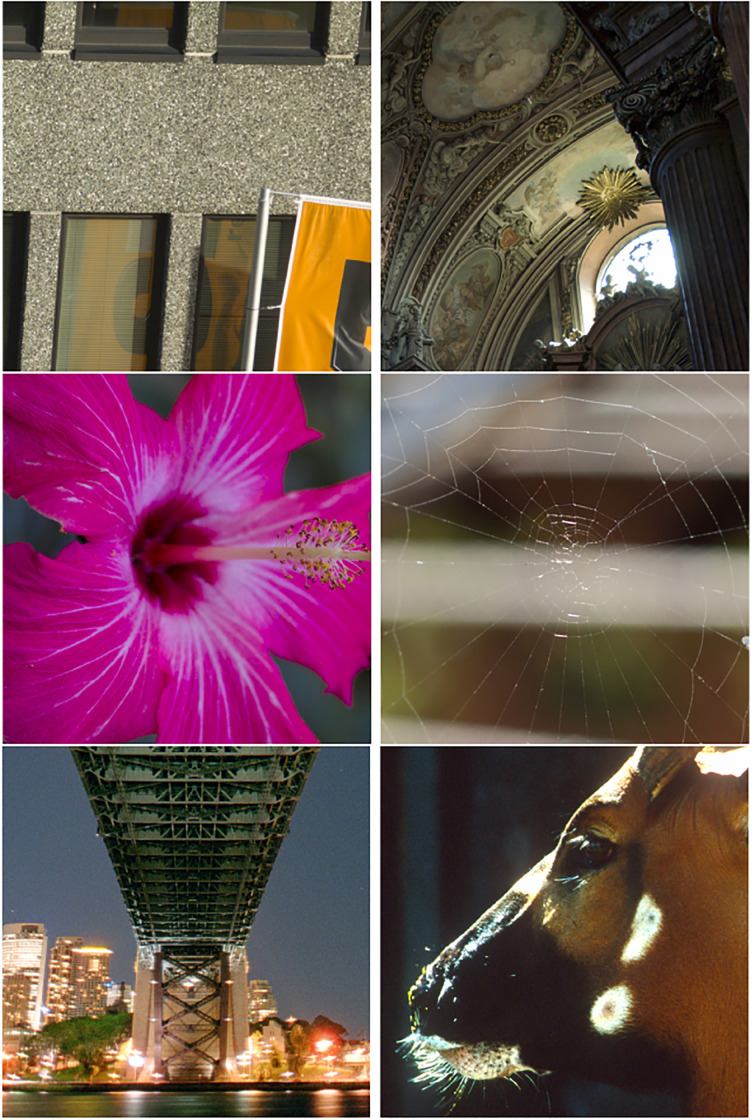
Illustration of the 1024 × 1024 × 3 panels from the six high resolution test images (No 10–15) listed in Table 4.

The relevance to image compression of the achieved sparsity by dct cross color transformation is illustrated in this section by comparison with results yielded by the compression standards JPEG, and WebP, on the 15 images in Table 4. These are typical images, used for compression tests, available in ppm or png format. The first 9 images are classic test images taken from [48]. The last six images are portions of 1024 × 1024 × 3 pixels shown in Fig 6 from very large high resolution images available on [49].

**Table 4:** Test images. The last column gives the approximation times to produce the results in Table 5.

No	Image	Size	time (secs)
1	Lenna	512 × 512 × 3	1.8
2	Goldhill	576 × 720 × 3	3.8
3	Barbara	576 × 720 × 3	3.2
4	Baboon	512 × 512 × 3	2.7
5	Zelda	576 × 784 × 3	2.5
6	Sailboat	512 × 512 × 3	1.8
7	Boy	512 × 768 × 3	3.8
8	Jupiter	1072 × 1376 × 3	2.9
9	Saturn	1200 × 1488 × 3	2.6
10	Building	1024 × 1024 × 3	9.5
11	Cathedral	1024 × 1024 × 3	7.6
12	Flower	1024 × 1024 × 3	3.6
13	Spider-web	1024 × 1024 × 3	3.8
14	Bridge	1024 × 1024 × 3	8.5
15	Deer	1024 × 1024 × 3	5.8

All the results have been obtained in the MATLAB environment (version R2019a), using a machine with CPU Intel(R) Core(TM) i7–3520M RAM 8GB CPU @ 2.90GHz. For the image approximation the HBW-OMP2D method was implemented with a C++ MEX file. All the channels and all the images were partitioned into blocks of size 16 × 16. The approximation times to produce the results in Table 5 are displayed in the last column of Table 4.

**Table 5:** Compression rate (bpp) corresponding to JPEG (bjp), WebP (bwb) and the proposed sparse representation (bsr), for the values of PSNR given in the 2nd column. The corresponding values of MSSIM are given in the 3rd to 5th columns. ssjp, sswb, and sssr indicate the MSSIM produced by JPEG, WebP and the sparse representation codec respectively.

I	dB	ssjp	sswb	sssr	bjp	bwb	bsr
1	35.9	0.98	0.98	0.97	3.28	2.21	**1.37**
2	36.6	0.98	0.98	0.98	3.63	2.47	**2.01**
3	37.2	0.98	0.99	0.99	3.67	2.80	**1.77**
4	28.8	0.97	0.97	0.96	5.80	4.66	**2.48**
5	39.3	0.98	0.98	0.95	2.61	1.81	**1.07**
6	30.9	0.98	0.98	0.95	4.49	3.21	**1.51**
7	32.6	0.97	0.98	0.97	4.34	3.07	**2.22**
8	48.2	0.99	0.99	0.99	0.60	0.51	**0.20**
9	49.0	0.99	0.99	0.99	0.34	0.36	**0.12**
10	37.4	0.99	0.99	0.99	3.41	2.35	**1.75**
11	38.5	0.99	0.99	0.99	2.84	1.83	**1.20**
12	41.5	0.99	0.99	0.99	1.78	1.08	**0.53**
13	45.0	0.99	0.99	0.99	1.55	1.10	**0.57**
14	34.5	0.99	0.99	0.99	3.57	2.48	**1.48**
15	30.9	0.97	0.97	0.96	3.76	2.59	**0.90**

For realizing the comparison we proceed as follows: we set the required value of PSNR as that produced by JPEG at quality = 95 and tune compression with the other methods to produce the same PSNR. In our codec the tuning is realized by approximating the image up to PSNR_*o*_ = 1.025 ⋅ PSNR (where PSNR is the targeted quality) and setting the quantization parameter Δ so as to reproduce the targeted value of PSNR.

For compression with JPEG we use the MATLAB imwrite command. The compression with WebP was realized using the software for Ubuntu distributed on [50]. All the approaches were tuned for producing the same value of PSNR as JPEG for quality 95. The Mean Structural SIMilarity (MSSIM) index [51] was then calculated with the approximation corresponding to those values of PSNR.

## 6 Conclusions

The application of a cross color transformation for enhancing sparsity in the atomic decomposition of RGB images has been proposed. It was demonstrated that the effect of the transformation is to re-distribute the most significant values in the dwt of the 2D channels. As a result, when approximating the arrays by disregarding the less significant entries, the quality of the reconstructed image improves with respect to disabling the cross color transformation. Four transformations have been considered: (i) a 3 point dct, (ii) the reversible YCbCr color space transform, (iii) the PC transform, (iv) a transformation learned from an independent set of images.

The quality of the image approximation was improved further by approximating the transformed arrays by an atomic decomposition using a separable dictionary and the greedy pursuit strategy HBW-OMP2D. The dct was singled out as the most convenient cross color transformation for approximating RGB color images in the wavelet domain.

The approximation approach has been shown to be relevant for image compression. By means of a simple coding strategy the achieved compression for typical test images considerably improves upon the most commonly used compression standards, namely JPEG and WebP.

## Supporting information

S1 Data (TXT)
